# Validated clinico-pathologic nomogram in the prediction of HER2 status in gastro-oesophageal cancer

**DOI:** 10.1038/s41416-019-0399-4

**Published:** 2019-02-12

**Authors:** Lorenzo Fornaro, Caterina Vivaldi, Annamaria Parnofiello, Clara Ugolini, Giuseppe Aprile, Giovanna De Maglio, Irene Pecora, Donatella Iacono, Francesca Crivelli, Silvia Catanese, Giovanni Gerardo Cardellino, Monica Lencioni, Enrico Vasile, Francesca Salani, Mario Clerico, Lorenzo Calvetti, Alfredo Falcone, Gianpiero Fasola, Gabriella Fontanini, Francesco Montagnani

**Affiliations:** 1 Unit of Medical Oncology 2, Azienda Ospedaliero-Universitaria Pisana, Pisa, Italy; 2 Department of Oncology, Azienda Sanitaria Universitaria Integrata di Udine, Udine, Italy; 3 Department of Laboratory Medicine, Section of Pathology, Azienda Ospedaliero-Universitaria Pisana, Pisa, Italy; 4 Department of Oncology, San Bortolo General Hospital, East District, Vicenza, AULSS8 Italy; 5 Department of Pathology, Azienda Sanitaria Universitaria Integrata di Udine, Udine, Italy; 6 Department of Oncology, Azienda Sanitaria Locale di Biella, Ponderano (BI), Italy; 7 Unit of Medical Oncology 1, Azienda Ospedaliero-Universitaria Pisana, Pisa, Italy; 8 Department of Surgical, Medical, Molecular Pathology and Critical Area, University of Pisa, Pisa, Italy

**Keywords:** Gastric cancer, Predictive markers

## Abstract

**Background:**

HER2 is the only validated predictive biomarker in gastro-oesophageal carcinoma (GOC). However, several factors, such as heterogeneity in protein expression, shortage of evaluable tumour tissue and need for quick target assessment, underline the usefulness of a pre-screening tool in order to anticipate HER2 status.

**Methods:**

Data from 723 consecutive GOC analysed for HER2 at four Italian Institutions were collected. HER2 positivity was defined as 3+ by immunohistochemistry (IHC) or 2+ with gene amplification by in situ hybridisation (ISH). A multivariate logistic regression model was built using data from 413 cases, whereas 310 patients served as validation cohort. C-index, visual inspection of the calibration plot, Brier score and Spiegelhalter *z*-test were used to assess the performance of the nomogram.

**Results:**

HER2 positive rate was 17.4%. Four variables were retained after adjustment in the final model: grading, Lauren’s histotype, pathologic material analysed (surgical specimen/biopsy) and site of tissue collection (primary tumour/metastases). Visual inspection of the calibration plot revealed a very good overlap between predicted and observed probabilities, with a Brier score of 0.101 and a non-significant Spiegelhalter *z*-test (*P* = 0.319). C-index resulted in 0.827 (95%CI 0.741–0.913).

**Conclusion:**

A simple nomogram based on always-available pathologic information accurately predicts the probability of HER2 positivity in GOC.

## Introduction

Gastro-oesophageal carcinoma (GOC) represents the third leading cause of cancer death worldwide,^1^ with a rising incidence of junctional cancers^2^ as well as tumours developing among younger individuals.^3^ In this tumour, the identification of new targets for effective treatment represents an unmet need.^4^ Up to now, the human epidermal growth factor receptor 2 (HER2) is the only available biomarker for personalised treatment in metastatic GOC. Trastuzumab demonstrated a significant overall survival benefit when added to first-line chemotherapy^5^ and is now approved for patients with HER2 overexpression at immunohistochemistry (IHC) or gene amplification by in situ hybridisation (ISH) in equivocal cases.^6^

Globally, the rate of HER2 positivity in GOC ranges from less than 10% to almost 50%, on the basis of the samples used for analysis and the laboratory techniques applied.^7^ Screening data from the ToGA trial has shown that several clinical and pathologic variables are associated with a higher rate of HER2 positivity, such as the location of the primary tumour in the gastro-oesophageal tract and Lauren’s subtype, HER2 positive cases being more prevalent among upper lesions and in cases with intestinal histology.^8^ Moreover, the authors reported a slightly higher rate of HER2 positive status for biopsies compared with surgical specimens.^8^ More recently, a meta-analysis reported that male gender, well or moderately differentiated as well as intestinal histology were all factors associated with higher rates of HER2 positivity in GOC.^9^ Other authors recently reviewed the available literature data partially confirming previous reports, but also underlining that large heterogeneity exists about the association between single clinico-pathologic markers and HER2 status.^10^

Considering the relevance of determining HER2 status in metastatic GOC for optimal treatment selection, the limitations linked to sample adequacy and methodology (type of test) used and the need for rapid HER2 report, we aimed to develop and validate a nomogram, based on easily accessible clinical or pathologic characteristics, which is able to anticipate the probability of harbouring a HER2 positive disease before direct tumour assessment by IHC and ISH.

## Materials and methods

### Sample identification and data collection

Consecutive GOC cases, analysed from January 2011 to December 2017 for HER2 status by IHC and ISH at the Department of Surgical, Medical, Molecular Pathology and Critical Area of the University of Pisa (Italy), were used as developing cohort. Findings in this subset were then confirmed in a separate, independent validation cohort of GOC cases analysed at independent Institutions in Italy since January 2011.

The following clinical and pathologic data were collected in both cohorts: gender (male *versus* female); primary tumour location (oesophago-gastric junction *versus* gastric body *versus* gastric fundus *versus* not specified); histologic subtype according to Lauren’s classification (intestinal *versus* diffuse *versus* not specified); tumour grading (G1 *versus* G2 *versus* G3 *versus* not specified); site of sampling (primary tumour *versus* metastasis); adequacy of pathologic material for analysis (inadequate material *versus* adequate material *versus* surgical specimen). Adequacy of biopsy for HER2 assessment was defined according to available evidence and recommendations, *i.e*. a number of at least 6 biopsies was considered as optimal for analysis.^11–14^

Pathologic features collected in the analysed datasets (tumour grading and Lauren’s subtypes) were evaluated by pathologists with high expertise in GOC. With regard to tumour grading, the WHO 2010 classification has been applied.^15^ According to this scale, G1 (well-differentiated) tumours are composed of well-formed glands, sometimes resembling metaplastic intestinal epithelium, whereas G3 (poorly-differentiated) tumours are composed of glands with highly irregular and atypical features that are recognised with difficulty. G2 (moderately-differentiated) tumours are indeed represented by neoplasms with intermediate features between well- and poorly-differentiated carcinomas.

### HER2 status assessment

HER2 status was initially assessed by IHC and in equivocal cases (i.e., 2+ at IHC) by ISH assays: HER2 positivity was then defined as IHC score of 3+ or 2+ with a positive ISH.

IHC was performed on formalin-fixed, paraffin-embedded tumour sections using the commercial antibodies PATHWAY antiHER-2/neu (4B5) Rabbit monoclonal (Roche-Ventana Medical Systems, Tucson, Az, USA). Sections were stained using automated slide stainer (Benchmark ULTRA, Roche Ventana Medical Systems, Tucson, Az, USA). An appropriate scoring system,^14^ also assimilated by the College of American Pathologists and regulatory authorities,^11^ exclusive for gastric tumours and accounting for type of specimen used (biopsy or surgical tissue), was applied for HER2 evaluation.

ISH assays were performed using the kit HER2 FISH pharmDx™ (DAKO/Agilent Santa Clara, CA United States) as *per* manufacturer’s instructions. Gene amplification by fluorescence ISH (FISH) was expressed as the ratio between the number of copies of the *HER2* gene and the number of copies of chromosome 17 within the nucleus counted in at least 20 cancer cells. The positivity of FISH was considered at a HER2:chromosome 17 ratio of ≥2.0. The entire specimens were screened for amplified regions at an x20 magnification. In borderline amplification cases (ratio within the range 1.8–2.2), 20 additional cells were re-counted.^11,14,16^

### Statistical analyses

Four hundred and thirteen patients in the development cohort were used to build an unconstrained logistic regression model able to predict HER2 positivity. First, all the variables were tested in univariate models. All statistically significant variables were then used to build multivariable models. Variables not significant, but with a strong literature support in favour of an association with HER2 status, were also considered for inclusion. Both backwards and forward method were used. Collinearity was evaluated using Fisher’s test, *t*-test and ANOVA, depending on the nature of the covariates, and Variance Inflation Factor (VIF). Global fit was evaluated with Nagelkerke’s R2, Somer’s D and model log-likelihood ratio chi-square. Final model was selected considering statistical significance of the covariates, the percentage of models in which it remained significant, their clinical plausibility and the global fit. A nomogram was then developed from the final model. Validation and calibration were performed on an external, independent dataset from three different Italian centres. C-index, visual inspection of the calibration plot, Brier score and Spiegelhalter *z*-test were used to assess the performance of the nomogram. The 95% confidence intervals of C-index were calculated with bootstrap method. A ROC curve was built with data from the validation set to assess sensitivity and specificity of the test at different cut-offs of predicted probabilities.

Package ‘rms' and ‘pROC' of R were used for all the analyses.

## Results

### Sample characteristics

Characteristics of the samples included in the two datasets are listed in Table 1. A total of 723 cases were included in the study, with 413 cases in the development cohort and 310 cases in the validation cohort. There were 70 (16.9%) and 56 (18.1%) HER2 positive cases in the development and validation cohorts, respectively. We observed significant differences between the two cohorts regarding the pathologic material used for analysis (i.e., higher number of biopsies compared to surgical specimens in the validation cohort) and primary tumour location (i.e., higher percentage of cases located in the gastric fundus in the validation cohort), whereas no differences were present in respect to all other collected characteristics. Since mixed tumours represented a minority (<3% of all cases) in both cohorts, tumour samples were reviewed and labelled as either intestinal or diffuse type according to the prevalent histotype in each case.

**Table 1:** Study cohort’s characteristics

	Development cohort (*N* = 413)		Validation cohort (*N* = 310)		
	*N*	(%)	*N*	(%)	*P*-value
HER2 status					0.695
Negative	343	(83.1)	254	(81.9)	
Positive	70	(16.9)	56	(18.1)	
Gender					0.237
Male	262	(63.4)	210	(67.7)	
Female	151	(36.6)	100	(32.3)	
Type of material					<0.001^a^
<6 biopsies	99	(24)	77	(24.8)	
≥6 biopsies	24	(5.8)	82	(26.5)	
Surgical sample	287	(69.5)	151	(48.7)	
Not specified	3	(0.7)	0	(0)	
Site of sampling					0.077
Primary tumour	365	(88.4)	287	(92.6)	
Metastases	48	(11.6)	23	(7.4)	
Primary tumour location					<0.001^a^
O-G junction	118	(28.6)	73	(23.5)	
Body	261	(63.2)	160	(51.6)	
Fundus	21	(5.1)	52	(16.8)	
Not specified	13	(3.1)	25	(8.1)	
Lauren’s histotype					0.559^a^
Intestinal	193	(46.7)	103	(33.2)	
Diffuse	183	(44.3)	108	(34.8)	
Not specified	37	(9)	99	(32)	
Tumour grading					0.343^a^
G1	10	(2.4)	11	(3.5)	
G2	100	(24.2)	56	(18.1)	
G3	289	(70)	178	(57.4)	
Not specified	14	(3.2)	65	(21)	

*N* number, *O–G* *junction* oesophago-gastric junction

^a^Not specified cases were excluded from the comparison

### Association of investigated variables with HER2 status and nomogram development

**Fig. 1:**
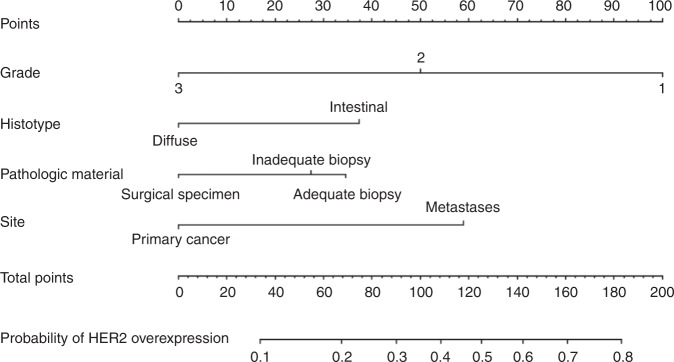
HER2 nomogram. Legend: Each variable is assigned a score in the ‘Points' axis. Locate the sum of all the single scores combined in the ‘Total Points' axis: the point identified by a line drawn downwards on the ‘Probability of HER2 overexpression' axis corresponds to the estimated probability of HER2 overexpression anticipated by the nomogram

When tested for association with HER2 positive status, the following features were confirmed significant at multivariate analysis in the development cohort: tumour grading (analysed as continuous variable), histotype (diffuse *versus* intestinal) and site of sampling (primary *versus* metastases). Type of pathologic material was not significant but, given the amount of data about its correlation with HER2 status, we retained this parameter in the multivariable model. A detailed list of univariate and multivariate analyses is given in Table 2. Site of primary cancer was also significant at univariate analysis but was excluded from the final model due to an excessive amount of collinearity with grading and histologic subtype. Exclusion of this variable did not affect the global fit, which remained good: *R*^2^ was 0.19, Somer’s D was 0.5, C-index was 0.75 and likelihood ratio chi-squared was 41.12 (*P* < 0.0001). Notably, we detected collinearity between grade and histologic type (*P* < 0.001). A slight collinearity was also present between pathologic material and site of sampling. On primary cancers the diagnoses were made by biopsy in 35.7% of the cases. On the contrary, the proportion of diagnoses made by biopsies on metastatic lesions was 66.7%. However, a formal Fisher test was negative (*P* = 0.144) and the VIF was always lower than 3 for all the variables tested, so it is unlikely that significant bias could be introduced. No first-degree interaction was significant. The definitive nomogram is depicted in Fig. 1.

**Table 2:** Association of clinical and pathologic factors with HER2 status: univariate and multivariate analyses

	Development cohort			
	Univariate		Multivariate	
	OR (95%CI)	*P*-value	OR (95%CI)	*P*-value
Gender				
Female	1		–	–
Male	0.60 (0.34–1.06)	0.076	–	
Type of material				
Surgical sample	1		1	
<6 biopsies	1.71 (0.96–3.05)	0.063	1.93 (0.91–4.11)	0.069
≥6 biopsies	2.47 (0.97–6.32)	0.058	2.29 (0.64–8.22)	0.201
Site of sampling				
Primary tumour	1		1	
Metastases	1.80 (1.03–4.15)	0.042	4.12 (1.11–15.2)	0.034
Tumour location				
Body	1		–	
O–G junction	1.88 (1.08–3.26)	0.023	–	–
Fundus	1.01 (0.28–3.59)	0.658	–	–
Histotype				
Intestinal	1		1	
Diffuse	0.23 (0.12–0.44)	<0.0001	0.41 (0.17–0.98)	0.045
Tumour grading				
G1 *versus* G2 *versus* G3	0.05 (0.02–0.16)	<0.0001	0.09 (0.02–0.41)	0.0018

*OR (95%CI)* odds ratio (95% confidence interval), *O–G junction* oesophago-gastric junction

### HER2 nomogram: external validation

**Fig. 2:**
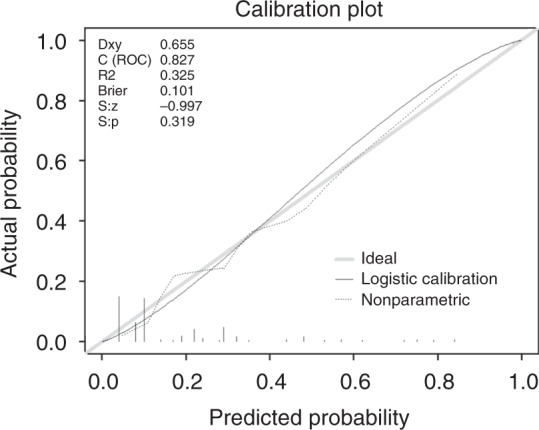
Calibration plot for external validation of the nomogram. Legend: Curves represent observed *versus* predicted probabilities, with gray line representing an ideal model (i.e., observed and predicted probabilities overlapping) and black and dotted lines representing the observed results. *Brier* Brier score, *C (ROC)* C-index, *Dxy* Somer’s D, *S:p* *P*-value of Spiegelhalter *z*-test, *S:z* *z*-value of Spiegelhalter *z*-test

Probabilities predicted by the nomogram were tested against those observed in the validation set. The nomogram discriminative ability was very good, with a C-index of 0.827 (95%CI 0.741–0.913). Brier score was 0.101 and the Spiegelhalter *Z*-test was not significant (*P* = 0.319). Visual inspection of the calibration plot showed a very good overlap between predicted and observed probabilities, with no relevant overestimations or underestimations (Fig. 2).

**Fig. 3:**
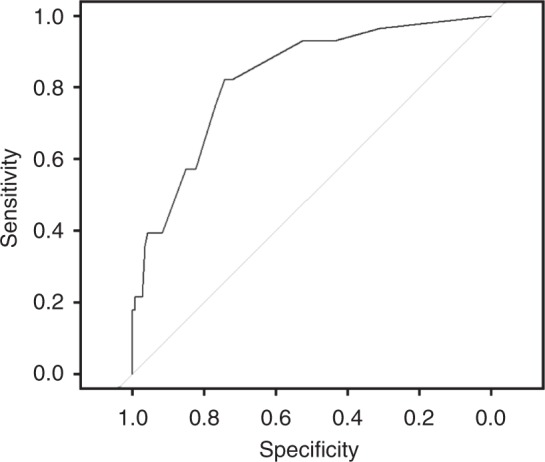
ROC curve between probabilities of HER2 positivity as predicted by the nomograms and actual HER2 status in the validation cohort. Legend: The curve represents the sensitivities and the specificities observed in the validation cohort at different cut-off values of probabilities predicted by the nomogram. Higher predicted probabilities correspond to higher values of sensitivity but lower values of specificity, and vice-versa

We developed a ROC curve on the validation cohort to calculate specificity and sensitivity of the test to detect the presence of HER2 positivity at different probabilities, as given by the model (Fig. 3). At a cut-off probability of 0.2, the model had 82% sensitivity and 74% specificity to detect the presence of HER2 positivity.

## Discussion

Defining HER2 status is crucial in the management of advanced GOC patients, as HER2 positive cases by IHC (and ISH, when required) may benefit from trastuzumab in combination with first-line chemotherapy.^5,6,17^ Missing HER2 positive tumours could then result in unexploited treatment opportunities, considering also that enrolment in clinical trials with novel anti-HER2 agents may be a suitable option. Therefore, in view of the relevance of this target (currently the only available validated predictive biomarker in GOC) and the challenges related to laboratory assessment of tumour samples in everyday practice,^18^ we developed and validated an easy-to-use and cheap nomogram, which predicts with high accuracy the chance of HER2 positivity before approved molecular diagnostics such as IHC and ISH are performed.^11^ Using a cut-off probability as given by the nomogram of 0.2, the test has a sensitivity of 0.82 and a specificity of 0.74 to detect the presence of HER2 positivity.

The variables included in the nomogram had been all associated with HER2 positive status, as it was previously found in available literature: lower tumour grading,^19^ intestinal histology,^19,20^ adequacy of analysed samples,^21^ and site of collection. Regarding the site of sampling (primary tumour *versus* metastases) larger heterogeneity is reported in different studies,^22–25^ ultimately confirming that specific clinico-pathologic features are not able to predict HER2 status when considered separately.^10^ In our series, tumour grading was the strongest predictor of HER2 status, in both cohorts. Both pathologic material and site of sampling were included in the model, moving from the evidence that biopsies are associated with higher probability of HER2 positive status compared with surgical specimens in our series. Similar findings were also reported by the ToGA trial investigators,^8^and may be justified by the different cut-off values used to define HER2 positivity. Another plausible explanation is that biopsies could be a surrogate variable, linked to deeper biological alterations associated with HER2 positivity. Again, there could be a potential overlap between biopsies and metastatic lesions, which are more likely to be HER2 positive. We indeed found in the developing set that a greater percentage of metastases are diagnosed by biopsies, introducing a slight collinearity in the model. However, the degree of such collinearity is small, unlikely to fully explain these findings. Our analyses also revealed a partial overlap between grading and Lauren’s subtype. However, the VIF for each parameter was always lower than 3, strongly suggesting that the model is not affected by relevant collinearity.

Adequate pathologic material for analyses is the key element of any quality-controlled laboratory procedure.^11,12,21,26^ Considering the heterogeneity in HER2 expression across tumour cells in GOC,^14^ a minimum of 5 biopsies are required according to literature data,^12,13,21^ with 6 to 8 specimens considered as the optimal threshold for adequate and reliable HER2 assessment in GOC by available recommendations.^11^ So, we set 6 as the reference number to categorise biopsy samples adequacy. Unfortunately, this issue has not been fully implemented in routine practice and our study confirms that a significant percentage of biopsies used for HER2 status assessment should have been considered indeed inadequate for appropriate evaluation. In our opinion, this nomogram could prompt clinicians to perform tumour re-biopsy in initially HER2 negative cases at molecular diagnostics but with an anticipated high probability of HER2 positive status and unavailability of sufficient tumour tissue for molecular analyses.^26^ As re-biopsy translates into increased risks for the patient and greater costs for health services, the tool we developed could be shared with all the specialties involved in the multidisciplinary management of GOC patients in order to raise awareness about the relevance of adequate sampling for optimal medical management.

Obviously, this nomogram cannot substitute the direct HER2 evaluation by IHC and ISH, according to approved diagnostics and guidelines.^11,14^ However, molecular tests are time consuming, may not be promptly accessible in all institutions and are subject to strict requirements with regards to the quality of biologic samples to be analysed. Therefore, clinicians could be interested in predicting HER2 status in the single patient at first assessment, in order to immediately evaluate different treatment options or study proposals in patients with higher probability of HER2 positive disease. This could also allow not delaying treatment initiation in patients with very low pre-test probability of harbouring a HER2 positive tumour. These patients could benefit from alternative chemotherapy regimens compared to the cisplatin plus fluoropyrimidine combination used in ToGA^5^: reasonable options are represented by a docetaxel-containing triplet, in order to increase activity and potentially efficacy,^27^ or oxaliplatin-based doublets, in order to improve safety.^28^

To conclude, HER2 status might be accurately and rapidly predicted by a simple nomogram based on four validated clinico-pathologic parameters. This tool could be easily implemented in clinical practice during the first assessment of the patient to add information for prompt case management.

## Data Availability

All data generated or analysed during this study are included in this published article.
